# Digital Twin-Based Intelligent Monitoring System for Robotic Wiring Process

**DOI:** 10.3390/s25195978

**Published:** 2025-09-26

**Authors:** Jinhua Cai, Hongchang Ding, Ping Wang, Xiaoqiang Guo, Han Hou, Tao Jiang, Xiaoli Qiao

**Affiliations:** 1 College of Mechanical and Electrical Engineering, Changchun University of Science and Technology, Changchun 130022, China; 2023200111@mails.cust.edu.cn (J.C.);; 2 Chongqing Research Institute of Changchun University of Technology, No. 5, Yulin Road, Yubei District, Chongqing 401120, China; 3 Avic Xi’an Aircraft Industry Group Co., Ltd., Xi’an 710089, China

**Keywords:** digital twin, robotic wiring, aerospace harness manufacturing, PSO-BPNN, adaptive servo gripper, kinematic optimization

## Abstract

In response to the growing demand for automation in aerospace harness manufacturing, this study proposes a digital twin-based intelligent monitoring system for robotic wiring operations. The system integrates a seven-degree-of-freedom robotic platform with an adaptive servo gripper and employs a five-dimensional digital twin framework to synchronize physical and virtual entities. Key innovations include a coordinated motion model for minimizing joint displacement, a particle-swarm-optimized backpropagation neural network (PSO-BPNN) for adaptive gripping based on wire characteristics, and a virtual–physical closed-loop interaction strategy covering the entire wiring process. Methodologically, the system enables motion planning, quality prediction, and remote monitoring through Unity3D visualization, SQL-driven data processing, and real-time mapping. The experimental results demonstrate that the system can stably and efficiently complete complex wiring tasks with 1:1 trajectory reproduction. Moreover, the PSO-BPNN model significantly reduces prediction error compared to standard BPNN methods. The results confirm the system’s capability to ensure precise wire placement, enhance operational efficiency, and reduce error risks. This work offers a practical and intelligent solution for aerospace harness production and shows strong potential for extension to multi-robot collaboration and full production line scheduling.

## 1. Introduction

The transformative wave of Industry 4.0 is fundamentally restructuring intelligent manufacturing paradigms. As a pivotal enabler, digital twin (DT) technology constructs high-fidelity virtual replicas of physical entities to achieve real-time production monitoring and dynamic optimization, thereby significantly boosting manufacturing efficiency and product quality [1,2]. Within robotic systems, DT’s value is particularly pronounced. By simulating operational environments to enable real-time synchronization between physical and virtual domains, it substantially reduces costly physical testing while enhancing motion precision and environmental adaptability [3,4,5]. Consequently, DT has been extensively implemented in complex automation processes, including welding, assembly, and milling, providing innovative workflow solutions [6,7,8,9]. Notably, in extreme hazard scenarios involving fire, explosions, or radiation, DT-AR integration establishes secure teleoperation platforms, allowing mission-critical operations to be performed from safe distances [10,11,12]. Essentially, DT converges AI and IoT technologies to deliver robust, data-driven support for robotic motion planning, process surveillance, and adaptive control through seamless cyber–physical interaction.

In aerospace manufacturing—a field demanding extreme precision and reliability—wire harness routing serves as the “neural network” of aircraft electrical systems and represents a critical manufacturing process [13]. However, this process has long relied heavily on manual operations, suffering from inefficiency, high error rates, and poor consistency [14]. With exponential growth in the complexity of modern aircraft electrical systems, wire harness requirements have surged dramatically; for instance, the Airbus A380 contains over 500,000 m of cabling. This enormous scale creates an urgent need for automation, where robotic systems demonstrate substantial application potential [15,16]. Recent years have witnessed the emergence of robotic arm-based automated routing: Nguyen [17] and Cho [18] focused, respectively, on 3D vision guidance and high-precision cable modeling, achieving automated production of small harnesses through dual-arm collaboration; Javier [19] developed a CAD-data-driven system for real-time robot trajectory generation during initial routing; and Song [20] addressed quality control with an AI-powered fault detection system that enhances inspection performance and cost-effectiveness. Nevertheless, fundamental challenges persist in manufacturing long-range, complex-configuration harnesses for large aircraft, including intricate kinematics coordination of seven-axis robots, insufficient intelligent manipulation of flexible cables by end-effectors, and lack of comprehensive digital process monitoring [21,22,23].

Although digital twin technology shows promise in robotics—e.g., Liu [24] optimized mobile robot trajectories using Unity, and Yun [25] calculated end-effector deviations via a five-dimensional model—its application remains unexplored for long-distance aviation routing scenarios. Current research exhibits gaps in aircraft harness manufacturing, including an absence of specialized control models for seven-axis coordination, intelligent clamping strategies tailored to cable properties, and DT frameworks with human–machine interaction solutions meeting long-distance routing demands [26], severely hindering automated production implementation.

To overcome these limitations, this research proposes a DT-based intelligent monitoring system for aerospace wire harnesses. The system employs a seven-axis configuration integrating a six-DOF robotic arm with linear translation axes, featuring a high-precision servo-electric gripper [27,28], and establishes a specialized architecture based on the five-dimensional DT framework (Physical Entity, Virtual Entity, Services, Twin Data, and Connections) [29]. This architecture deeply integrates kinematic planning, adaptive clamping, and virtual–physical interaction modules to achieve closed-loop optimization throughout the routing process. Core innovations manifest in the following three aspects: (1) developing a seven-axis coordinated motion model minimizing cumulative joint displacement to reduce long-path routing energy consumption while enhancing stability; (2) establishing a harness-specific 5D architecture-based twin mapping paradigm, with real-time physical–virtual data interaction enabled via OPC UA, that ensures real-time synchronization between physical and virtual layers while enabling pre-routing simulation; and (3) introducing an innovative PSO-BPNN (Particle Swarm Optimization—Back Propagation Neural Network) algorithm for adaptive fixture control. Compared with conventional PID control, this algorithm enables intelligent, predictive adjustment of clamping force based on anticipated cable performance, thereby preventing damage while ensuring precise wiring.

System validation covers multiple dimensions: the virtual simulation platform pre-validates trajectories and layout parameters before physical execution, drastically reducing actual wiring errors; the seven-axis robot motion planning proves highly efficient and rational; and the PSO-BPNN clamping mechanism optimizes cable placement quality through autonomous parameter tuning. Experiments demonstrate that the system significantly improves routing efficiency and precision, delivering an innovative yet practical solution for aerospace harness manufacturing.

The paper is structured as follows: Section 2 details the five-dimensional DT architecture design; Section 3 elaborates on seven-axis kinematics, PSO-BPNN clamping control, and virtual–physical interaction; Section 4 validates system performance via prototype experiments; and Section 5 concludes the study and outlines future directions.

## 2. Digital Twin Model Architecture

**Figure 1:**
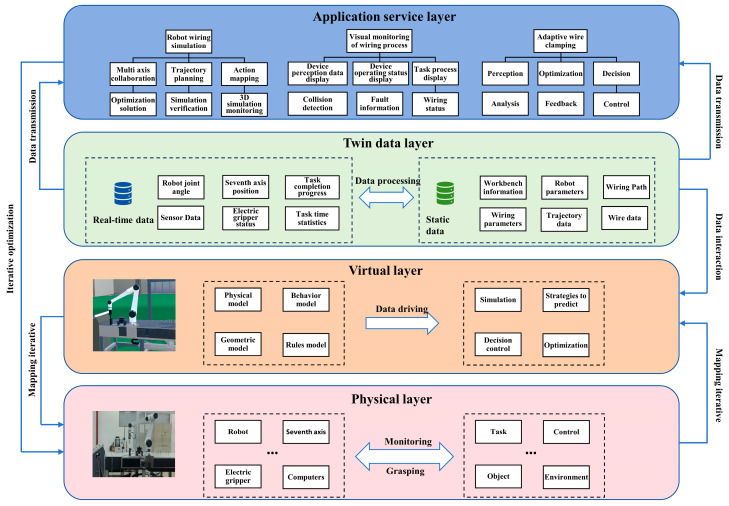
Five-dimensional architecture of the digital twin system for robotic wiring.

As an advanced solution, the digital twin system achieves real-time interaction between virtual and physical robots through network communication, integrating sensing technologies to synchronize twin data from both domains. Leveraging this twin data, the system accomplishes real-time motion mapping, trajectory planning verification, wiring process simulation, control parameter optimization, and 3D visual monitoring—establishing comprehensive management of robotic wiring operations. Building upon Prof. Tao’s five-dimensional DT model [29], we develop a dedicated 5D architecture for intelligent monitoring of flexible wiring robots, as illustrated in Figure 1. The architecture comprises the following five core components: a Physical layer, Virtual layer, Twin data layer, Application Service layer, and their interconnecting communication channels.

(1) Physical Layer

The Physical Layer incorporates a robotic arm, seventh axis (linear rail), controller, workbench, servo-electric gripper, sensors, and data acquisition system. Multi-sensory and operational data collected from physical robots creates and drives their virtual counterparts.

(2) Virtual Layer

**Figure 2:**
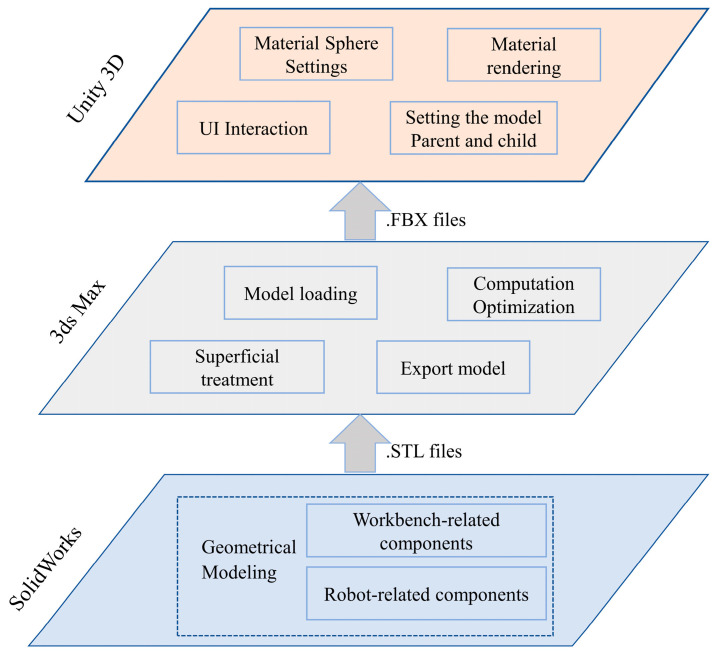
Process of building the digital twin model.

This layer mirrors physical entities in cyberspace through quad-domain mapping (physical properties, behavioral logic, geometric features, and operational rules), accurately representing position, morphology, and kinematic characteristics to enable operation simulation and monitoring. Constructing a wiring robot’s digital twin involves establishing physical rules, developing geometric representations, and creating monitoring scenarios, as shown in Figure 2. Leveraging Unity3D2022’s robust 3D visualization, flexible development environment, superior interactivity, and exceptional extensibility, we create near-photorealistic monitoring scenes where real-time rendering enables rapid construction of high-precision models and realistic simulations. Geometric twin modeling initiates with SolidWorks2024-based 3D design, producing structurally and parametrically identical digital replicas of physical robots. Models exported as STL undergo texture mapping and material rendering in 3ds Max, and are then converted to FBX format for Unity3D integration. Within Unity3D, components are assembled according to kinematic relationships, forming a comprehensive virtual robot model. Robot kinematics principles are then applied to establish inter-joint motion relationships, defining constraints including rotation angles and velocities to enable precise physical behavior emulation.

(3) Twin Data Layer

Twin data encompasses information reflecting entity states and behaviors, categorized as static data and real-time data. Static data comprises robotic geometric configurations, motion envelopes, and spatial relationships that remain constant post-initialization. Real-time data encompasses operational metrics generated during runtime, including task completion progress, gripper status, operation duration, six-axis joint angles, and seventh-axis position/velocity/acceleration, collectively mirroring live robotic states. Collection and processing of both data types enables high-fidelity mapping between virtual and physical entities. Within Unity3D, the DT system establishes Oracle database connections via SQL queries, enabling real-time ingestion of multi-source heterogeneous data acquired through socket communication from physical environments. Using Unity’s XCharts plugin, this data undergoes dynamic visualization through graphical plots and textual displays in the UI dashboard.

Moreover, production-state data enables not only real-time DT monitoring, but also permanent archival in information systems, facilitating process traceability and post hoc analysis—effectively transforming DT into a production playback mechanism. Post-production analysis via DT technology achieves high-precision process reconstruction, establishing a robust foundation for manufacturing optimization and managerial enhancement.

(4) Service Layer

Integration of the Physical, Virtual, and Twin Data layers establishes a DT application platform for wiring robots within the visualization software. Platform capabilities include robotic motion planning, wiring process simulation, dynamic data visualization, and adaptive wire clamping strategy optimization. During wiring operations, the system controls actuators and processes sensor signals to visualize routing activities, enabling intelligent planning, monitoring, and management, which enhances harness placement precision and quality.

(5) Connectivity

Connectivity serves as the communication bridge between all components. Socket communication with custom protocols is implemented throughout the system, where modules encapsulate data into packets (header + payload) before transmission. Packet headers contain metadata descriptors. Socket interfaces bridge all layers, encapsulating TCP/IP APIs to provide data channels that enable cross-application information exchange via sockets, enhancing communication synergy. These APIs construct robust links between physical robots and virtual environments, enabling bidirectional cyber–physical interaction.

## 3. Methodology

Within the wiring robot’s digital twin system, the development and integration of motion simulation constitute the primary tasks of the Service Layer, encompassing the following three aspects: robotic motion planning, intelligent control of the end-effector (electric gripper), and simulation with visual monitoring of the wiring process.

### 3.1. Kinematic Planning for Wiring Robot

#### 3.1.1. Model Construction

**Figure 3:**
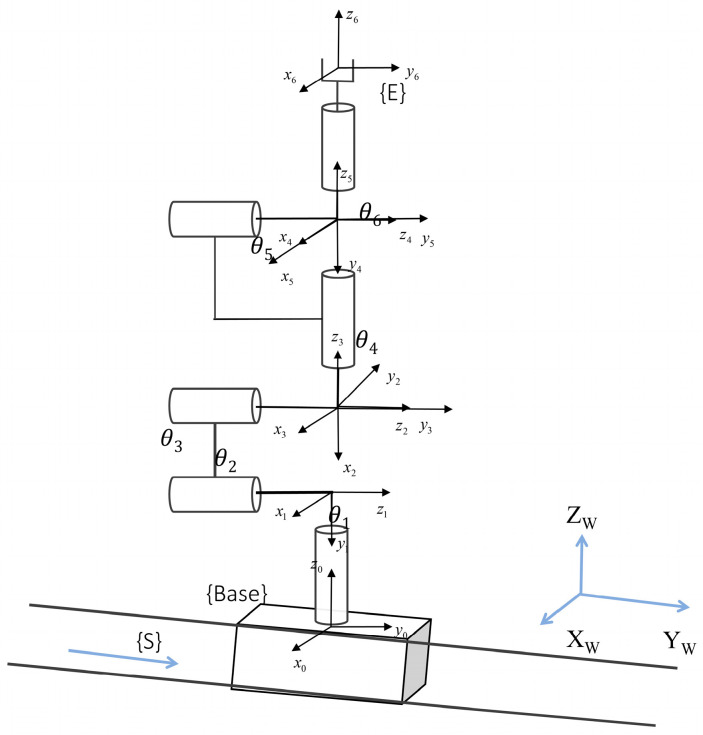
Coordinate systems and DH model of the robotic arm and end-effector.

Physical model construction involves mapping the kinematic structure of the robotic wiring system onto its virtual counterpart. Each key component in virtual space possesses its own coordinate system; defining the transformation relationships between these coordinate frames is essential to simulate the robotic wiring system’s motion structure. To address the spatial coverage requirements of aerospace harness routing tasks, the system adopts a 7-DOF redundant configuration integrating a 6-axis collaborative robot arm with a linear rail, establishing a unified kinematic model based on the Denavit–Hartenberg (D-H) convention. The coordinate frames and D-H model are depicted in Figure 3, where the global reference frame {W} has its origin at the rail’s zero point, with its $Z_{W}$ vertically upward and $Y_{W}$ aligned with the rail direction; the rail coordinate frame {S} shares its origin with {W}; the manipulator base frame {B} is fixed to the sliding carriage, translating with the rail displacement variable $d_{7}$; joint coordinate frames are established according to the standard D-H convention; and the end-effector frame {E} is located at the flange center point.

The D-H parameters for the 7-DOF system are listed in Table 1, comprising the external linear rail axis (Joint 0) and the six rotary axes of the manipulator body (Joints 1–6), where *d_i_* denotes link offset, *a_i_* is link length, *α_i_* represents link twist, and *θ_i_* is the joint variable.

For Joint 0 (rail), $d_{7}$ serves as the displacement variable, with a travel range of 0–4400 mm, meeting long-distance wiring requirements; in Joint 1, the base height parameter *d*_1_ = 200 mm combined with a link twist *α*_1_ = π2 establishes a 90° axial transformation from $Z_{0}$ to $Z_{1}$; and Joints 4–6 utilize an alternating αi = ±90° configuration to achieve a spherical wrist structure, causing the rotation axes of J4 and J6 to intersect at the wrist center point. This parametric model satisfies Pieper’s criterion, providing the theoretical foundation for closed-form inverse kinematics.

#### 3.1.2. Forward Kinematics of the 7-Axis Robot

System pose mapping is achieved through cascading coordinate transformations. The base translation transformation caused by the linear rail motion is as follows:

(1) TBW=100d7010000100001

where $d_{7}$ is the rail displacement (mm). This transformation represents a pure translation of the base along the $Y_{W}$. The manipulator body kinematics employ the standard D-H model. The general transformation formula between adjacent coordinate frames $\left\{J_{\left\{i-1\right\}}\right\}$ and $\left\{J_{i}\right\}$ is as follows:

(2) Tii−1=cosθi−sinθicosαisinθisinαiaicosθisinθicosθicosαi−cosθisinαiaisinθi0sinαicosαidi0001

where $\theta_{i}$ is the joint variable (rad), $\alpha_{i}$ is the link twist (rad), $a_{i}$ is the link length (mm), and $d_{i}$ is the link offset (mm). The complete forward kinematics equation from the world coordinate frame to the end-effector is as follows:

(3) TEW=TBW⋅TEB=REpE01

where $p_{E}={\left[p_{x},p_{y},p_{z}\right]}^{T}$ represents the position vector of the end-effector in 3D space and $R_{E}\in SO(3)$ is the orientation rotation matrix. This model precisely describes the pose (position and orientation) of the cable end in 3D space, providing the mathematical foundation for wiring trajectory generation. In aerospace harness wiring tasks, $p_{E}$ (Position of End-effector) precisely controls the placement location of the cable, while $R_{E}$ (Rotation of End-effector) determines the mating orientation of the connector.

#### 3.1.3. Inverse Kinematics of the 7-Axis Robot

For the 7-degree-of-freedom (DOF) redundant system, this paper proposes a hierarchical optimization-based inverse kinematics (IK) solving strategy.

Based on the task-space decomposition principle, the target pose TE,desW=Rdes,pdes is transformed to the base coordinate frame as follows:

(4) TE,desB=Rdespdes−[d7*,0,0]T01

where $d_{7}^{*}$ is the optimized linear rail displacement (mm), and $p_{\mathrm{des}}$ is the target position in the world coordinate frame (mm).

A slide rail position optimization model is constructed as follows:

(5) $$\min_{d_{7}}{\left\|J_{a}^{†}\left(p_{\mathrm{des},y}-d_{7}\right)\right\|}^{2}+\lambda {\left\|\theta -\theta_{\mathrm{mid}}\right\|}^{2}\;s.t.\;d_{7}\in \left[0,L_{s}\right]$$

where $J_{a}^{†}$ is the Moore–Penrose pseudoinverse (mm/rad) of the translational part of the manipulator Jacobian matrix, $p_{\mathrm{des},y}$ is the $Y_{W}$ -component of the desired end-effector position (mm), $\theta_{\mathrm{mid}}$ is the mid-range joint angle vector (rad), λ=0.1 is a weighting coefficient, and Ls is the maximum stroke of the rail (mm). This model minimizes the joint motion of the manipulator, reducing mechanical wear and extending lifespan, while improving motion accuracy and stability and decreasing energy consumption during the wiring process.

Leveraging the three intersecting wrist axes property (coaxial J4/J6), an analytical solution is employed.

Calculation of wrist center position is performed as follows:

(6) $$p_{w}=p_{\mathrm{des},B}-d_{6}\cdot R_{\mathrm{des}}\left[\begin{array}{c} 0\\ 0\\ 1 \end{array}\right]$$

where $p_{\mathrm{des},B}$ is the target position in the base frame (mm), and $d_{6}$ = 100 mm is a D-H parameter.

Joints 1–3 are solved via geometric analysis, as follows:

(7) $$\left\{\begin{array}{ll} \theta_{1} & =\mathrm{atan}2(p_{w,y},p_{w,x})\\ r & =\sqrt{p_{w,x}^{2}+p_{w,y}^{2}},\;h=p_{w,z}-d_{1}\\ \theta_{3} & =\arccos\left(\frac{r^{2}+h^{2}-a_{2}^{2}-a_{3}^{2}}{2a_{2}a_{3}}\right)\\ \theta_{2} & =\mathrm{atan}2(h,r)-\mathrm{atan}2(a_{3}\sin\theta_{3},a_{2}+a_{3}\cos\theta_{3}) \end{array}\right.$$

where $d_{1}=200\;\mathrm{mm}$, $a_{2}=250\;\mathrm{mm}$, $a_{3}=300\;\mathrm{mm}$ represent relevant D-H parameters, r is the horizontal projection distance of the wrist center (mm), and h is the height of the wrist center (mm).

The attitudes of Joints 4–6 are decomposed via ZYZ Euler angles, as follows:

(8) R63=(R30)TRdesθ4,θ5,θ6=ZYZ-EulerR63

where R30 is the rotation matrix determined by the configuration of Joints 1–3, and ZYE denotes the Z-Y-Z Euler angle decomposition function.

Feasible solutions are finally screened through the optimization criteria, as follows:

(9) $$q^{*}=\arg\underset{q_{k}}{\min}[\kappa (J(q_{k}))+\gamma \|q_{k}-q_{p}rev\|]$$

where κ(J) is the condition number of the Jacobian matrix, γ = 0.5 is the continuity weight coefficient, and $q_{p}rev$ is the joint vector at the previous moment. This strategy significantly reduces the trajectory interruption rate in aviation wiring looping and knotting tasks, thereby improving wiring efficiency.

### 3.2. Adaptive Wire Clamping

In automatic aircraft wiring systems, the interaction between the robotic gripper and the cable significantly affects wiring accuracy. Due to fluctuations in cable diameter and micro-scale contact dynamics, the friction behavior exhibits strong nonlinearity. While direct pressure sensing offers high accuracy, it cannot be practically integrated into the gripper’s compact structure. As an alternative, motor current feedback provides a feasible means for indirect force estimation and better aligns with the system’s design constraints.

Experimental results show that cable diameter and gripper opening exhibit a strong correlation with current (*R*^2^ > 0.85), justifying their selection as input features for the predictive model. A Backpropagation Neural Network (BPNN) is, thus, constructed, using these geometric parameters to estimate the motor current and implicitly reflect the clamping state.

To enhance model robustness and avoid local minima during training, a Particle Swarm Optimization (PSO) algorithm is employed to optimize the initial weights and biases of the BPNN. The optimization process follows the update rules

(10) $$X_{i}^{t+1}=X_{i}^{t}+\omega V_{i}^{t}+c_{1}r_{1}(p_{best}-X_{i}^{t})+c_{2}r_{2}(g_{best}-X_{i}^{t})$$

**Figure 4:**
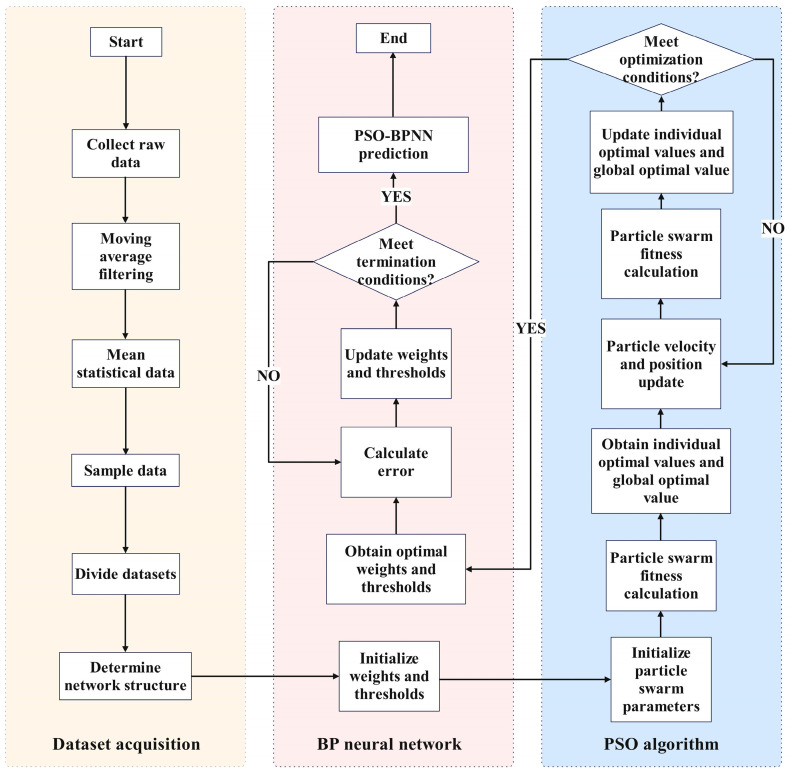
PSO-optimized BPNN process flow.

In this formulation, $V_{i}^{t}$ and $X_{i}^{t+1}$ represent the velocity and position of particle *i* at iteration *t*, while $p_{best}$ and $g_{best}$ denote its personal best and the global best positions found by the swarm. The parameters ω, $c_{1}$, and $c_{2}$ control inertia and learning influence, and the random variables $r_{1}$ and $r_{2}$ introduce stochastic diversity to improve exploration. Through iterative updates, particles dynamically converge toward optimal solutions, improving the BPNN’s generalization ability. Once trained, the optimized model enables real-time estimation of motor current from wiring parameters, guiding adaptive gripper adjustments throughout the wiring task, as illustrated in Figure 4.

### 3.3. Virtual–Physical Interaction in the Wiring Process

#### 3.3.1. Wiring Process Simulation

Dynamic simulation serves as a critical pre-execution rehearsal for the aircraft wiring process. After the wiring path is planned at the application service layer, the inverse kinematics of the robotic arm are computed to derive joint motion parameters based on the trajectory of the end-effector. These parameters are then validated and refined through dynamic simulation to ensure safe and rational wire routing.

In this process, the Unity3D platform plays a central role, providing a digital twin environment that replicates the robot’s physical behavior with high fidelity. The digital twin not only visualizes the wiring process in real time, but also accelerates simulation through high-speed rendering, reducing debugging time and facilitating process iteration and optimization. This virtual production environment also supports layout planning and production scheduling in a closed-loop simulation framework.

To ensure safety during wiring—particularly on high-precision workbenches densely populated with guiding pillars—real-time collision detection is implemented. Based on Unity 3D’s physics engine, colliders are added to critical components such as the robotic arm, end-effector, workbench, and pillar models. Mesh colliders are applied to high-risk parts like the last three joints and the end-effector, enabling precise collision detection via surface meshes. For parts with lower collision risk, simplified box or capsule colliders are used to reduce computational cost.

#### 3.3.2. Visualization and Human–Machine Interaction

The digital twin-driven robotic wiring system incorporates a multimodal human–machine interface (HMI), enabling immersive interaction and real-time monitoring. Through Unity3D’s multi-view UI capabilities, users can visualize the robot’s motion status and process states across different perspectives.

**Figure 5:**
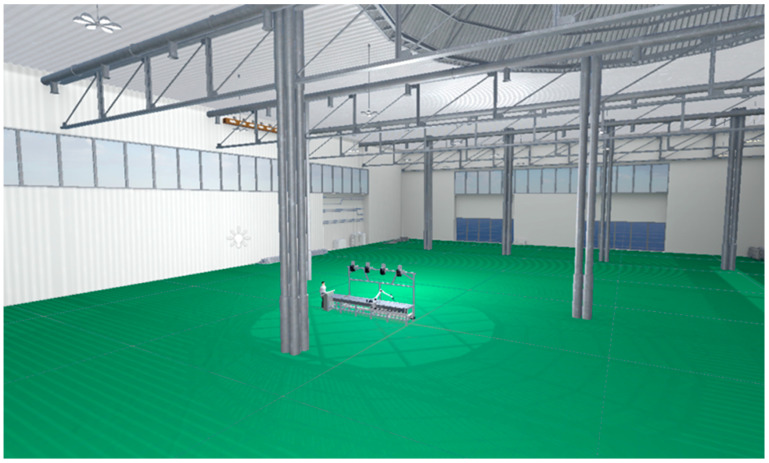
Scene in Unity showing light source implementation.

Interaction is facilitated via embedded controls such as buttons and text fields, which dynamically respond to user inputs and trigger corresponding events. Input devices such as a mouse or keyboard allow users to navigate the scene by adjusting the camera’s position, angle, and zoom level. To enhance immersion and realism, lighting effects are refined by adjusting directional and point light sources. For instance, as shown in Figure 5, a directional light simulates sunlight, while two point lights evenly illuminate the workbench, enhancing material texture and depth perception across virtual components.

#### 3.3.3. Digital Twin Mapping Mode

The cyber–physical mapping mechanism lies at the core of the digital twin model, enabling real-time synchronization between physical wiring robots and their virtual counterparts. Physical systems collect multidimensional data—such as motor current, joint angles, and wiring positions—through multisensor fusion. These data are transmitted across platforms via the OPC UA protocol and used to drive the virtual model.

**Figure 6:**
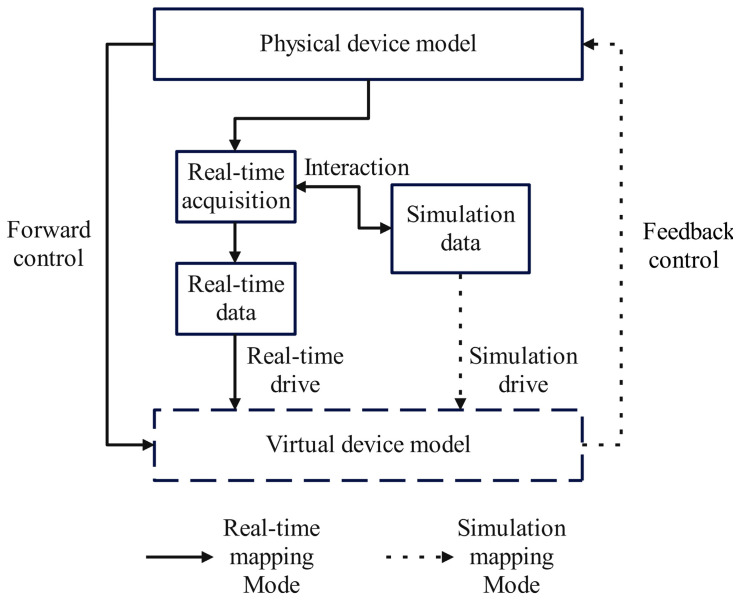
Virtual–physical mapping mode of digital twin.

The following two mapping modes are supported: real-time mode and simulation mode, as illustrated in Figure 6. In real-time mode, live physical data continuously update the virtual model, enabling bidirectional interaction where physical measurements like joint angles or currents actively control the virtual system—referred to as “physical-to-virtual control”. In contrast, simulation mode uses predefined process parameters to guide virtual execution. After physical execution, sensor feedback is compared with the simulation database to identify deviations, which are then used to optimize the process parameters. Updated instructions are transmitted via OPC UA back to the physical controller, completing the “virtual-to-physical control” loop.

For example, if the motor current of the end-effector exceeds a preset threshold, an emergency stop is triggered to prevent cable damage. Similarly, when the virtual system detects a critical proximity between the robot and nearby obstacles—indicating a collision radius has been breached—it issues a stop command with low latency to ensure system safety.

## 4. Experiments and Results

### 4.1. Digital Twin Prototype of the Robotic Wiring System

**Figure 7:**
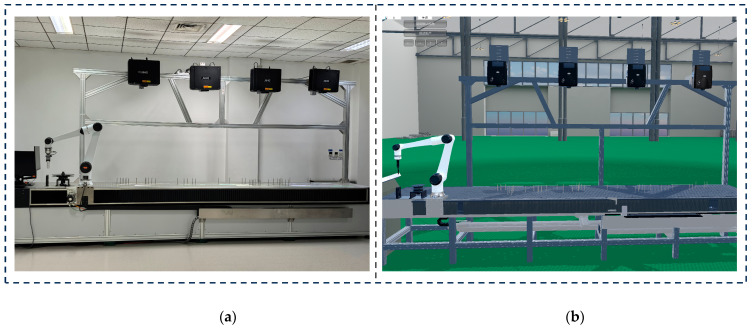
Digital twin prototype of the robot wiring system: (**a**) Physical entity. (**b**) Digital twin model geometry.

The constructed digital twin prototype platform for the robotic wiring system is shown in Figure 7a, and its geometric model is presented in Figure 7b. The digital twin interaction platform is deployed on an industrial PC (IPC) configured with a 13th Gen Intel(R) Core(TM) i7-13700H processor (2.40 GHz), NVIDIA GeForce RTX 4060 Laptop GPU, and 16 GB of RAM, and establishes a communication link with a Siemens PLC via PROFINET Ethernet. The PLC connects in real time to the Han’s Robot controller through the MODBUS TCP protocol, configuring the robot’s seventh axis as an external axis directly scheduled by the controller to achieve coordinated motion control between the robot body and the mobile base. Socket communication is employed for the interface between the robot and the digital twin system. Additionally, the wiring end-effector gripper and other auxiliary devices are connected to the PLC via the fieldbus, enabling force and position parameter configuration of the gripper to control wiring friction and ensure high-quality harness placement on the workbench.

### 4.2. Wiring Quality Prediction Based on the PSO-BP Algorithm

After completing robot position calibration, wiring experiments are conducted to collect data samples for quality prediction and parameter optimization. A full-factorial experimental design is employed, with raw data processed using a sliding average filter and mean statistical analysis. All input features are normalized to the range [0, 1] using MinMax scaling to ensure comparability during model training. The cable diameter ranges from 0.5 mm to 3.5 mm (increment: 0.3 mm, 11 levels), and the gripper opening ratio ranges from 10% to 100% (increment: 10%, 10 levels), resulting in 110 initial parameter combinations.

Samples where the motor current exceeds 55 mA—indicating cable damage—are considered outliers and removed (12 cases), leaving 98 valid data sets. Stratified sampling is applied to split the dataset into a training set (78 samples) and a testing set (20 samples), ensuring consistent distributions of cable diameter and gripper opening in both sets. Stratified sampling is adopted to maintain the balance of feature distributions between training and testing sets, given the limited sample size. The BPNN architecture consists of an input layer with 2 neurons (for cable diameter and gripper opening), one hidden layer containing 10 neurons with ReLU activation, and an output layer with a single neuron using linear activation. The Adam optimizer is used with a learning rate of 0.01, and the network is trained for 500 epochs. No explicit regularization is applied due to the small dataset size.

To improve predictive accuracy, Particle Swarm Optimization (PSO) is applied to optimize the initial weights of the Backpropagation Neural Network (BPNN). The PSO parameters are set as follows: swarm size of 50 particles, maximum of 200 iterations, learning factors $c_{1}$ = 1.5 and $c_{2}$ = 1.7, and an inertia weight that decreases linearly from 0.9 to 0.4.

**Figure 8:**
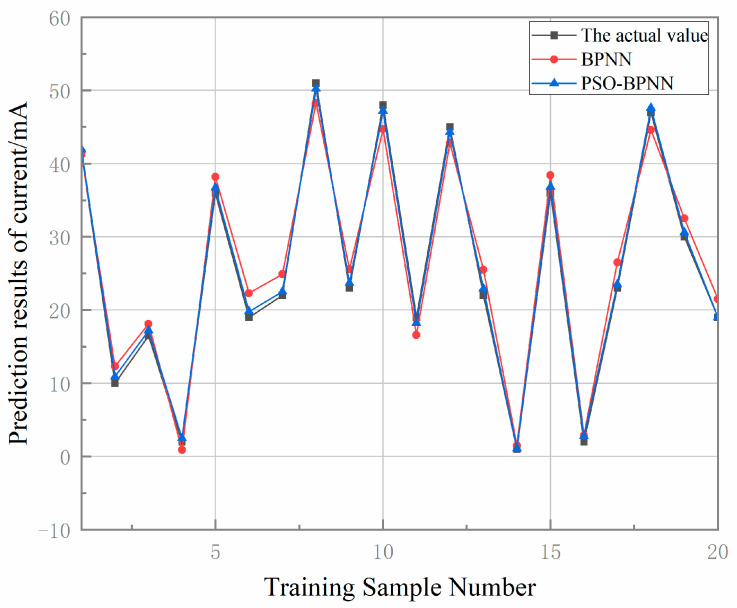
Current prediction results from various models.

**Figure 9:**
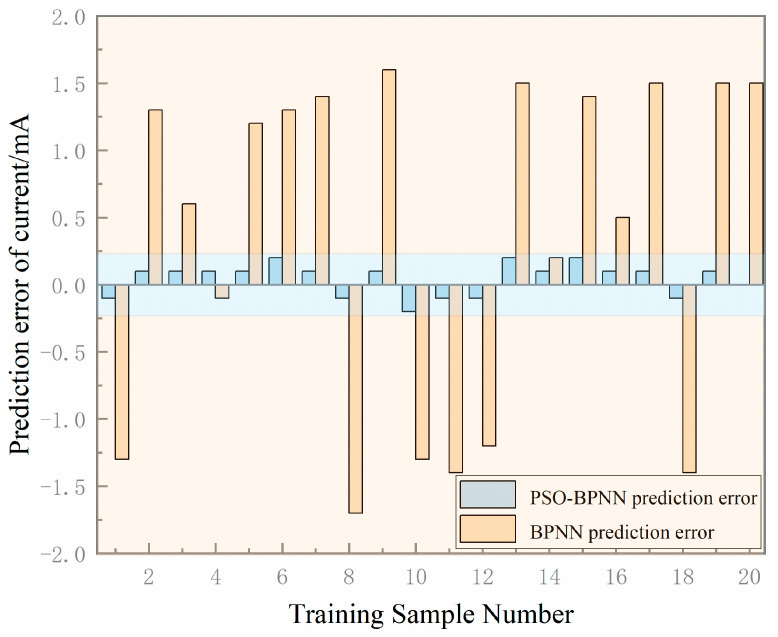
Prediction errors for current from different models.

A comparison of predicted versus measured gripper motor currents is shown in Figure 8 and Figure 9. For both figures, the *x*-axis (Training Sample Number) ranges from 1 to 20, and the *y*-axis range is consistent across all data series in each figure to ensure valid comparison. The BPNN model exhibits a prediction error range of 0.2 mA to 1.7 mA, whereas the PSO-BPNN model achieves an error range of 0 to 0.2 mA—representing an 88.24% improvement in maximum prediction accuracy. The number of training samples N used in both experiments is 20.

Model evaluation metrics, summarized in Table 2, show that for motor current prediction, the PSO-BPNN model achieves reductions of 52.11% in RMSE, 63.22% in MAPE, and 54.78% in MAE compared to the standalone BPNN, with the coefficient of determination (R2*R*2) improving to 0.981. To assess the uncertainty of the results, all metrics (R^2^, RMSE, MAE, MAPE, and Maximum ERROR) are obtained by repeating the training and evaluation procedure five times with different random seeds, and they are reported as mean ± standard deviation. The average network inference time per prediction cycle is 2.5 ms. Error analysis indicates that the standalone BPNN suffers from significant prediction deviations, whereas PSO-BPNN demonstrates close agreement between predicted and measured values. These results confirm that the PSO-BPNN model offers a superior fitting performance and generalization capability, making it an effective tool for wiring quality prediction.

### 4.3. Simulation and Visual Monitoring Verification

The application service layer of the digital twin system performs path planning based on the current wiring task, generating the trajectory for a single wiring operation and converting it into executable code. Prior to physical execution, a full simulation of the wiring process is carried out in Unity3D to validate the feasibility and accuracy of all parameters and execution results. Only after successful verification is the physical wiring task executed.

**Figure 10:**
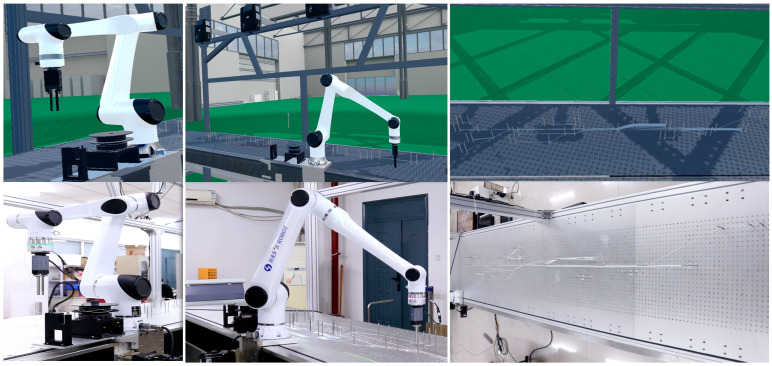
Comparison between robot wiring simulation and physical object.

To evaluate the effectiveness of the proposed wiring method, an assembly experiment is conducted using a sample harness blueprint. Figure 10 presents the simulation and real-world execution results of the robotic automatic wiring process, including wire pickup, routing, and the complete deployment of the wire segments according to the blueprint. As shown, the physical and virtual environments maintain a high degree of visual consistency. The entire cable path planned in the virtual space is faithfully replicated during the physical wiring process on a 1:1 scale. Moreover, operators can obtain a real-time, non-contact view of the physical operation status through the robotic wiring system. Specifically, host–robot communication is implemented at a sampling rate of 50 Hz, with an end-to-end latency below 250 ms. While this configuration is sufficient for the current experimental setup, further optimization of hardware and control loop frequency may be required to meet stricter real-time operational demands in future applications.

**Figure 11:**
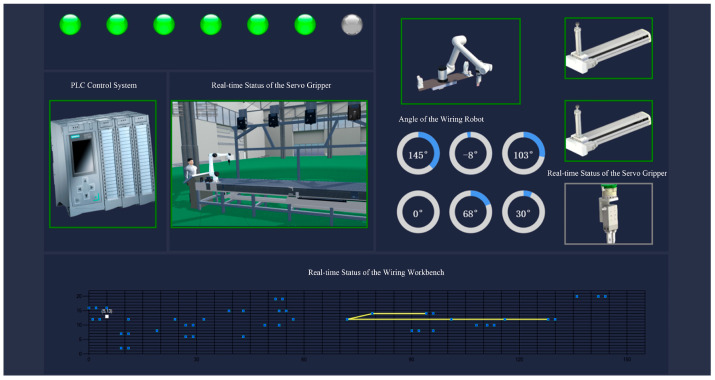
Visualization interface.

As depicted in Figure 11, the 3D visualization module of the robotic wiring digital twin system combines various elements such as text fields, dynamic charts, virtual environments, and robot representations. This Unity3D-based interface displays dynamic information collected from sensors and physical devices, such as robotic joint motion parameters, device operational status, communication interface status, and the current wiring trajectory. Through continuous chart updates, the system enables real-time data analysis and statistical evaluation, which efficiently track the robot’s performance state and the progression of the wiring process.

The experimental results show that the proposed digital twin system enables seamless cyber–physical interaction, allowing for accurate simulation and 3D visualization of the wiring process. The findings confirm that the system delivers realistic scene rendering, smooth motion simulation, and low-latency data acquisition and transmission. These capabilities facilitate effective real-time monitoring of the physical wiring process and demonstrate the system’s potential for practical deployment in industrial applications.

## 5. Conclusions

This study successfully developed a digital twin-based robotic wiring monitoring system tailored for aerospace harness manufacturing. By deeply integrating physical entities with virtual models, the system enables intelligent coordination and full-process control of the wiring operation. Furthermore, the system significantly improves wiring efficiency, reduces labor intensity, and ensures that the wiring quality meets the requirements of practical applications.

In the physical layer, a seven-axis robotic system equipped with an adaptive servo gripper accurately performs wire laying tasks on a positioning-pin workbench. In the virtual layer, a five-dimensional digital twin architecture is established, forming a closed-loop simulation framework that encompasses motion planning and quality prediction. The core innovation of the system lies in the establishment of a bi-directional interaction loop—“virtual guiding reality, and reality optimizing virtuality.” The digital twin-driven pre-execution simulation significantly reduces on-site commissioning risks, while the industrial Ethernet-based cyber–physical synchronization ensures high-fidelity trajectory reproduction. Furthermore, the PSO-BPNN-based intelligent gripper control model adaptively learns wire characteristics, thereby maintaining high-quality harness placement throughout.

Experimental validations confirm that the proposed system exhibits strong engineering applicability in complex wiring tasks. Its 3D visualization interface provides intuitive decision-making support to operators, enhancing transparency and operational efficiency.

Despite these promising results, the system still faces certain limitations, particularly in its adaptability to challenging environments. For instance, wiring stability issues may arise when handling larger-diameter cables or complex structures such as twisted pairs. In this study, the evaluation was conducted primarily on a single-cell/robot configuration, and its scalability to multi-robot cells remains to be validated. Additionally, domain shift challenges may occur when applying the system to different cable types, coatings, or friction conditions, potentially affecting performance. The gripper current model also relies on certain assumptions that may not hold across all scenarios, which could limit its generalizability. Future research will aim to address these challenges by enhancing the system’s sensing and adaptive capabilities, enabling more robust and intelligent responses to variable wiring scenarios, while also exploring the potential of multi-robot collaboration and full production line integration under the digital twin framework to further improve manufacturing scalability and efficiency.

## Figures and Tables

**Table 1:** D-H parameters for the wiring robot.

Link I	*α_i_*/(Rad)	*d_i_*/(mm)	*a_i_*/(mm)	*θ_i_*/(°)	Range
0	0	*d*_7_	0	0	0–4400 mm
1	π/2	200	0	*θ*_1_	−360° to +360°
2	0	0	250	*θ*_2_	−135° to +135°
3	0	0	300	*θ*_3_	−153° to +153°
4	−π/2	150	0	*θ*_4_	−360° to +360°
5	π/2	0	0	*θ*_5_	−180° to +180°
6	−π/2	100	0	*θ*_6_	−360° to +360°

**Table 2:** Model prediction performance analysis.

Index	BPNN	PSO-BPNN	Change
R^2^	0.924 ± 0.012	0.981 ± 0.008	+6.17%
RMSE	1.42 ± 0.05	0.68 ± 0.03	−52.11%
MAE	1.15 ± 0.04	0.52 ± 0.02	−54.78%
MAPE	8.7 ± 0.3	3.2 ± 0.1	−63.22%
Maximum ERROR	1.7 ± 0.1	0.2 ± 0.05	−88.24%

## Data Availability

The data that support the findings of this study are available from the correspondence upon reasonable request.
